# Serum MicroRNA profile in patients with colon adenomas or cancer

**DOI:** 10.1186/s12920-017-0260-7

**Published:** 2017-04-20

**Authors:** Yajie Zhang, Min Li, Yijiang Ding, Zhimin Fan, Jinchun Zhang, Hongying Zhang, Bin Jiang, Yong Zhu

**Affiliations:** 1 Central Laboratory, The Third Affiliated Hospital of Nanjing University of Chinese Medicine, Nanjing, People’s Republic of China; 2 Clinical Biobank of Nanjing Hospital of Traditional Chinese Medicine, Nanjing, People’s Republic of China; 3 National Medical Centre of Colorectal Disease, The Third Affiliated Hospital of Nanjing University of Chinese Medicine, Nanjing, People’s Republic of China; 4 Department of Oncology, The Third Affiliated Hospital of Nanjing University of Chinese Medicine, Nanjing, People’s Republic of China; 5 Department of Clinical Laboratory, The Third Affiliated Hospital of Nanjing University of Chinese Medicine, Nanjing, People’s Republic of China; 6 Department of Pathology, The Third Affiliated Hospital of Nanjing University of Chinese Medicine, Nanjing, Jiangsu 210001 People’s Republic of China

**Keywords:** Serum miRNAs, Adenomas, Colon cancer

## Abstract

**Background:**

Colon cancer, one of the most common causes of cancer-related deaths, arises from adenomatous polyps. In these years, circulating microRNAs (miRNAs) have attracted increasing attention as novel biomarkers for colon cancers. The dysregulated circulating miRNAs in patients with colon adenomas has not been well-understood.

**Methods:**

Here, we aimed to identify miRNA profile in the serum of patients with colon adenomas or colon cancer by using microarray. Then we validated eight differentially expressed miRNAs (DEMs) by qRT-PCR and predicted their targets.

**Results:**

We identified 26 DEMs from Adenomas versus Normal comparison (11 up-regulations and 15 down-regulations), 72 DEMs from Cancer versus Normal comparison (19 up-regulations and 53 down-regulations) and 17 DEMs from Cancer versus Adenomas comparison (4 up-regulations and 13 down-regulations). Moreover, three DEMs identified from Cancer versus Normal comparison were included in the list of DEMs identified from Cancer versus Adenomas comparison, and may be specific diagnostic biomarkers for colon cancer. Five down-regulated miRNAs identified from Cancer versus Normal comparison were included in the list of DEMs identified from Adenomas versus Normal comparison, and may be important for the development of colon polyps and cancer.

**Conclusions:**

We discovered 8 circulating miRNAs associated with colon adenomas and colon cancer, and these miRNAs may potentially serve as noninvasive screening biomarkers for colon cancer. Our study is useful for expanding our understanding in the development of colon adenomas and colon cancer, and thus provide novel insights into colon cancer pathogenesis and prevention.

**Electronic supplementary material:**

The online version of this article (doi:10.1186/s12920-017-0260-7) contains supplementary material, which is available to authorized users.

## Background

Colon cancer is the third most common cause of cancer-related deaths in the world, with an estimated incidence of 1,000,000 new cases and a mortality of more than 600,000 deaths each year [1]. Colon cancer arises from mucosal colonic polyps, which has the two most common histologic types: hyperplastic and adenomatous. Epidemiologic, clinical, pathologic and molecular evidence indicates that all colon cancers arise from adenomas [2]. Early diagnosis and cancer prevention with polypectomy can reduce the mortality of colon cancer. Therefore, more efficient diagnostic tools for early detection would help improve patients’ survival [3]. Colonoscopy, currently the preferred screening method for colon cancer, is invasive, inconvenient, and expensive, whereas the fecal occult blood test (FOBT), another screening method, has the limitation of relatively low sensitivity and specificity [4]. Consequently, there is an urgent need for identifying new diagnostically sensitive, specific, and noninvasive markers to improve the early detection of colon cancer.

microRNAs (miRNAs) are endogenously expressed small non-coding RNAs, 18 to 25 nucleotides in length [5]. More than 3000 miRNAs have been discovered in plants, animals and viruses. miRNAs can down-regulate the expression of their target genes through binding to the 3′ untranslated region of their target mRNAs, and thus play an important function in many cellular processes such as cell differentiation, proliferation, and apoptosis [5]. Recently, altered expression of miRNAs has been reported in various human cancers [6, 7], including colon cancer [8–11]. Since Mitchell et al. highlighted the presence of miRNAs in plasma [12], circulating miRNAs have gained much attention because they are highly stable and easily obtained through noninvasive procedures [11, 13–17]. miRNA profiles have been investigated in blood from colon cancer patients [11, 15–17]. However, a limited number of studies have been undertaken searching for dysregulated miRNAs in blood of patients with colon adenomas [18].

The aim of this study was to identify miRNA profile in the serum of patients with colon adenomas or colon cancer. Serum samples from patients with colon adenomas or colon cancer and from healthy subjects were assessed for miRNA profile by using miRCURY LNA™ microRNA Array system, which contains 3100 capture probes. We characterize dysregulated serum miRNAs in colon adenomas or cancer that may serve as a new non-invasive approach in detection of colon adenomas and colon cancer.

## Methods

### Study design and patient samples

This study was divided into two phases: phase I, miRNA profiling; and phase II, validation by quantitative RT-PCR (qRT-PCR). Human blood samples were obtained from National Medical Centre of Colorectal Disease and stored at Clinical Biobank of Nanjing Hospital of Traditional Chinese Medicine. All patients and healthy persons had signed informed consent for donating their samples to Clinical Biobank of Nanjing hospital of traditional Chinese medicine. Ethical approval for the project was received from the Nanjing Hospital of Traditional Chinese Medicine Ethics Committee (project reference, KY2015003, KY2015005, KY2015020). Whole blood was collected from the participants, separated into serum within two hours and then stored in -80 °C for later use. All the serum sample was added with 10:1(v:v) RNAlater (Ambion, Austin, TX). For miRNA profiling, three patients with colon adenoma (male; age 62 ± 5 years), three patients with colon cancer (male; age 60 ± 2 years) and three sex and age-matched healthy subjects (age 59 ± 2 years) were enrolled. For validation of microarray data, serum was collected from an independent group of 20 patients with colon adenoma (11 male; age 64 ± 6 years), 20 patients with colon cancer (12 male; age 65 ± 4 years) and 20 sex and age-matched healthy subjects (11 male; age 60 ± 6 years).

### The blood sample selection

All blood samples were collected before any therapeutic procedures, including surgery, chemotherapy, and radiotherapy. All the patient and health persons was examined by colonoscopy to diagnose whether they have any colon disease. Exclusion criteria included inflammatory bowel disease, a family history of familial adenomatous polyposis or hereditary non-polyposis colon cancer or previous colonic surgery. The biopsies were sectioned using a cryostat microtome and hematoxylin-cosin stain slides were evaluated for tumor content by a pathologist. (median tumor content in the samples was 50%, range 30–80%). After tumor resection, resected specimens were processed routinely for histopathological assessment at the time of surgery and classified according to the Tumor Node Metastasis (TNM) staging system. Three samples of adenoma or cancer patients was selected to miRNA profile. Their tumor localization was one right colon, one transverse colon, and one left colon, respectively. Health person whose sex and age-matched was taken as control. MiRNA screening samples and 60 validated samples was selected randomly from the patient recruited from The Third Affiliated Hospital of Nanjing University of Chinese Medicine in 2015. Clinical and histopathological characteristics of the colon adenoma patients, colon cancer patients and health controls was list in Table 1.

**Table 1:** Clinical and histopathological characteristics of the colon adenoma patients, colon cancer patients and health controls

Characteristics		Health	Adenoma	Cancer	*P* value
Sex	Male	11	11	12	0.934
	Female	9	9	8	
Age	≤60	10	10	12	0.765
	>60	10	10	8	
Histological grade	Low	–	20	8	0.00
	Middle or high	–	0	12	
Tumor invasion depth (T)	T1/T2	–	20	9	0.00
	T3/T4	–	0	11	
Lymph node metastasis (N)	Yes	–	0	12	0.00
	No	–	20	8	
TNM stage	I/II	–	20	9	0.00
	III/IV	–	0	11	

### RNA extraction

Total RNA containing small RNA was extracted from serum specimens using miRNeasy Serum/Plasma Kit (QIAGEN, Valencia, CA, USA) according to according to manufacturer’s instructions, which efficiently recovered all RNA species, including miRNAs. The quality and quantity of extracted RNA was determined by using Nanodrop spectrophotometer (Nanodrop Technologies, Wilmington, Delaware, USA).

### MicroRNA profiling of serum specimens

Profiling was performed using miRCURY LNA™ microRNA Array system (Exiqon, Vedbaek, Denmark), which contains 3100 capture probes, covering all human, mouse, and rat microRNAs annotated in miRBase 18.0. One microgram of each sample was 3′-end-labeled with Hy3^TM^ fluorescent label using miRCURY™ Hy3™/Hy5™ Power labeling kit (Exiqon) as recommended by the manufacturer. Briefly, the mixture was incubated for 30 min at 37 °C, and was terminated by incubation for 5 min at 95 °C. Then 3.0 μL of labeling buffer, 1.5 μL of fluorescent label (Hy3^TM^), 2.0 μL of DMSO, 2.0 μL of labeling enzyme were added into the mixture. The labeling reaction was incubated for 1 h at 16 °C, and terminated by incubation for 15 min at 65 °C. Hybridization of the microarray slides were performed as recommended by Exiqon. After washing and drying, the hybridized slides were scanned the Axon GenePix 4000B microarray scanner (Axon Instruments, Foster City, CA). Scanned images were then imported into GenePix Pro 6.0 software (Axon) for grid alignment and data extraction. Replicated miRNAs were averaged and miRNAs with intensities ≥30 in all samples were chosen for calculating normalization factor. Expressed data were normalized using median normalization. After normalization, significant differentially expressed miRNAs (DEMs) were identified through Volcano Plot filtering. Hierarchical clustering was performed using MEV software (v4.8, TIGR).

### MiRNA quantification by quantitative RT-PCR (qRT-PCR)

To validate the microarray data, we measured the expression levels of selected DEMs by using SYBR green qRT-PCR assay. In brief, 30 ng of serum RNA containing miRNA was polyadenylated by poly(A) polymerase and reverse transcribed to cDNA using miScript Reverse Transcription kit (QIAGEN, Valencia, CA, USA) following the manufacturer’s instructions. Real-time qPCR was performed using miScript SYBR Green PCR kit (QIAGEN) in ABI 7500 Real-time PCR system (Applied Biosystems; Foster City, CA, USA). The miRNA-specific primer sequences for qRT-PCR were designed based on the miRNA sequences obtained from the miRBase database (http://microrna.sanger.ac.uk/) and listed in Table S1. Each sample was run in triplicates for analysis. The expression levels of miRNAs were normalized to *C. elegans* miR-39 (miRNeasy Serum/Plasma Spike-In Control, QIAGEN, Cat. No. 219610). Statistically significant differences were determined using one-way-ANOVA test. *P < 0.05, **P < 0.01, ***P < 0.001.

## Results

### Identification of differentially expressed miRNAs (DEMs)

**Fig. 1:**
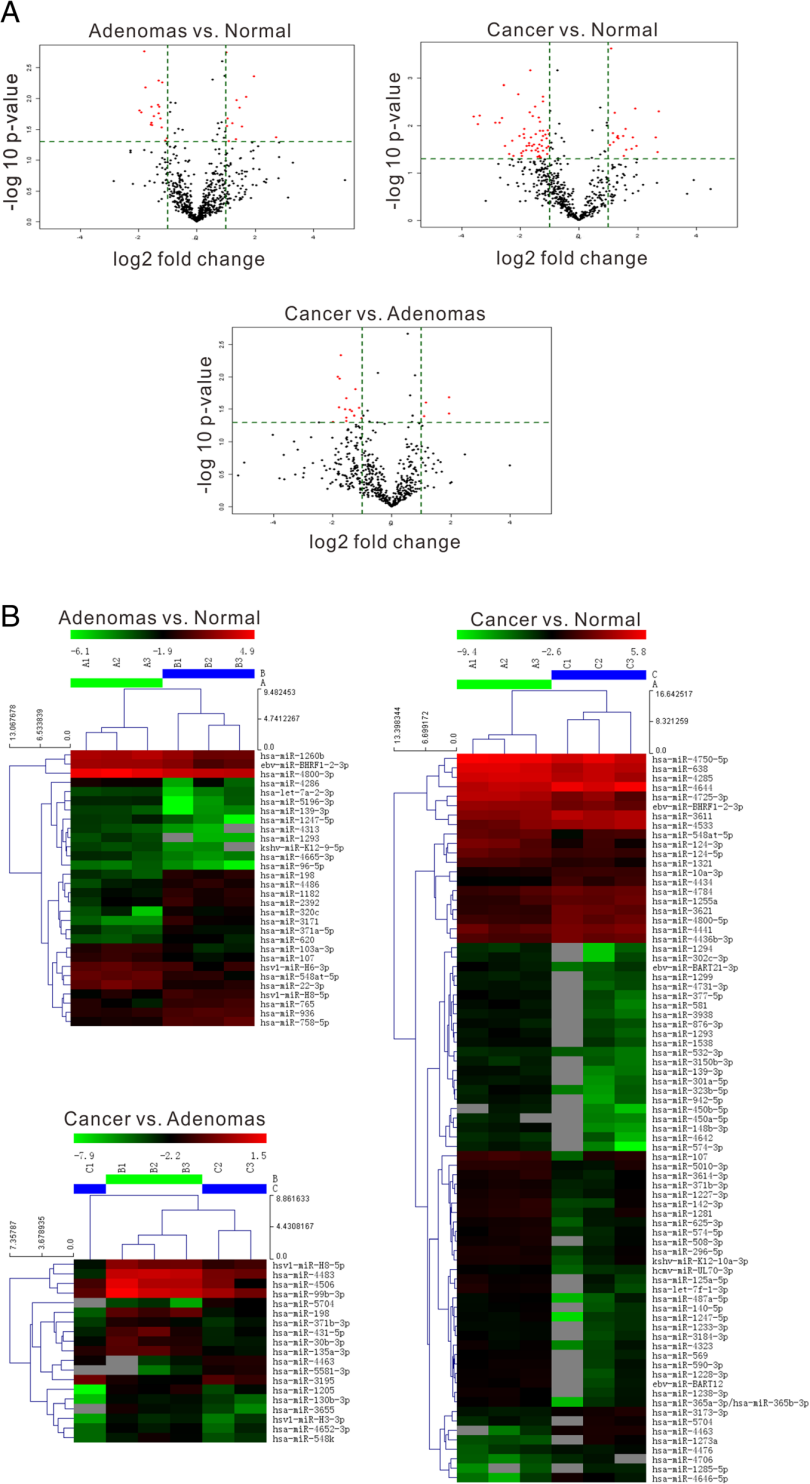
miRNA expression profiles of serum specimens from patients with colon adenoma (Adenomas, group B) or colon cancer (Cancer, group C) and healthy subjects (Normal, group A). **a** The volcano plots of DEMs between Adenomas and Normal, Cancer and Normal, and Cancer and Adenomas. The vertical lines correspond to 2.0-fold up and down, respectively, and the horizontal line represents a *P*-value of 0.05. So the red point in the plot represents the DEMs with statistical significance. **b** The hierarchical clustering analysis of DEMs is shown. See also Additional file 1: Tables S1-S3

To systematically determine differences in miRNA expression in the serum specimens of patients with colon adenomas or colon cancers and healthy individuals, the expression levels of 3100 miRNA were examined using the miRCURY LNA™ microRNA Array system. After filtering low intensity miRNAs, raw signal intensities were normalized by median. miRNAs that passed Volcano Plot filtering (Fold Change ≥ 2.0, *P*-value ≤ 0.05; Fig. 1a) were defined as DEMs and hierarchical clustering analysis (Fig. 1b) was performed. The result of hierarchical clustering shows distinguishable miRNA expression profiling among samples.

In the present study, we identified a total of 26 significant DEMs with 11 up-regulations and 15 down-regulations in colon adenoma samples (Adenomas, group B), when compared with healthy samples (Normal, group A) (Table 2). The most DEMs were detected between the colon cancer (Cancer, group C) and healthy controls, with 19 up-regulated and 53 down-regulated in the serum from colon cancer patients (Table 3). Seventeen miRNAs, including 4 up-regulations and 13 down-regulations (Table 4), were dysregulated in the serum of patients with colon cancer when compared to the serum of patients with colon adenomas. These DEMs were functionally related with cancer cell migration and invasion (miR-10a [19, 20], miR-124-3p [21, 22], let-7f [23], miR-140-5p [24], miR-139-3p [25] and miR-302c-3p [26]), growth (miR-124-3p [22], miR-124-5p [27] miR-140-5p [24] and miR-302c-3p [26]), cell cycle regulation (miR-107 [28]), cell apoptosis (miR-139-3p [25]), chemoresistance (miR-140-5p [29] and miR-487a [30]), and DNA repair (miR-638 [31]), and thus may be more critical to the development of colon cancer.

**Table 2:** DEMs identified from Adenomas (B) vs. Normal control (A) comparison

	ID	Fold change	*P*-value	FDR
Up-regulated	hsa-miR-3171	6.61	0.0431	0.7509
	hsa-miR-198	3.89	0.0044	0.6526
	hsa-miR-758-5p	3.25	0.0095	0.6526
	hsa-miR-320c	2.93	0.0287	0.6577
	hsa-miR-4486	2.77	0.0142	0.6526
	hsa-miR-1182	2.57	0.0106	0.6526
	hsa-miR-2392	2.54	0.0458	0.7509
	hsa-miR-765	2.33	0.0251	0.6526
	hsa-miR-371a-5p	2.13	0.0493	0.7660
	hsa-miR-620	2.08	0.0213	0.6526
	hsa-miR-936	2.03	0.0018	0.6526
Down-regulated	hsa-miR-22-3p	0.26	0.0157	0.6526
	hsa-miR-1293	0.27	0.0170	0.6526
	hsa-miR-548at-5p	0.29	0.0017	0.6526
	hsa-miR-5196-3p	0.30	0.0067	0.6526
	hsa-miR-4286	0.34	0.0266	0.6526
	hsa-miR-139-3p	0.34	0.0136	0.6526
	hsa-miR-4313	0.34	0.0249	0.6526
	hsa-miR-1247-5p	0.35	0.0272	0.6548
	hsa-miR-103a-3p	0.36	0.0195	0.6526
	ebv-miR-BHRF1-2-3p	0.40	0.0127	0.6526
	hsa-miR-107	0.40	0.0052	0.6526
	hsa-miR-4665-3p	0.41	0.0175	0.6526
	hsa-miR-4800-3p	0.41	0.0136	0.6526
	hsa-miR-1260b	0.42	0.0211	0.6526
	hsa-miR-96-5p	0.43	0.0299	0.6729
	hsa-let-7a-2-3p	0.50	0.0447	0.7509

**Table 3:** DEMs identified from Cancer (C) vs. Normal control (A) comparison

	ID	Fold change	*P*-value	FDR
Up-regulated	hsa-miR-4463	6.57	0.0050	0.2162
	hsa-miR-4646-5p	6.42	0.0362	0.2704
	hsa-miR-1273a	6.16	0.0178	0.2424
	hsa-miR-1285-5p	3.87	0.0269	0.2620
	hsa-miR-3621	3.78	0.0044	0.2162
	hsa-miR-4434	3.58	0.0178	0.2424
	hsa-miR-4706	3.46	0.0310	0.2692
	hsa-miR-4533	3.06	0.0327	0.2704
	hsa-miR-4800-5p	3.01	0.0117	0.2424
	hsa-miR-4644	2.88	0.0434	0.2899
	hsa-miR-1255a	2.84	0.0166	0.2424
	hsa-miR-3611	2.52	0.0185	0.2424
	hsa-miR-5704	2.51	0.0167	0.2424
	hsa-miR-3173-3p	2.48	0.0177	0.2424
	hsa-miR-4441	2.32	0.0054	0.2184
	hsa-miR-4436b-3p	2.27	0.0225	0.2547
	hsa-miR-4784	2.23	0.0144	0.2424
	hsa-miR-10a-3p	2.13	0.0002	0.2162
	hsa-miR-4476	2.08	0.0256	0.2620
Down-regulated	hsa-miR-574-3p	0.08	0.0064	0.2325
	hsa-miR-148b-3p	0.09	0.0093	0.2334
	hsa-miR-450b-5p	0.10	0.0062	0.2325
	hsa-miR-139-3p	0.14	0.0088	0.2334
	hsa-miR-323b-5p	0.15	0.0088	0.2334
	hsa-miR-942-5p	0.16	0.0070	0.2325
	hsa-miR-301a-5p	0.17	0.0014	0.2162
	hsa-miR-450a-5p	0.18	0.0269	0.2620
	hsa-miR-124-3p	0.19	0.0396	0.2866
	hsa-miR-581	0.21	0.0211	0.2537
	hsa-miR-1294	0.21	0.0435	0.2899
	hsa-miR-3938	0.24	0.0022	0.2162
	hsa-miR-377-5p	0.24	0.0168	0.2424
	hsa-miR-302c-3p	0.26	0.0381	0.2819
	hsa-let-7f-1-3p	0.26	0.0437	0.2899
	hsa-miR-4642	0.27	0.0345	0.2704
	hsa-miR-1247-5p	0.27	0.0152	0.2424
	hsa-miR-4725-3p	0.27	0.0119	0.2424
	hsa-miR-142-3p	0.28	0.0181	0.2424
	hsa-miR-3150b-3p	0.29	0.0332	0.2704
	hsa-miR-487a-5p	0.29	0.0271	0.2620
	hsa-miR-1293	0.31	0.0263	0.2620
	hsa-miR-4731-3p	0.32	0.0053	0.2184
	hsa-miR-532-3p	0.32	0.0007	0.2162
	hsa-miR-140-5p	0.32	0.0178	0.2424
	hsa-miR-4323	0.33	0.0341	0.2704
	hsa-miR-365a-3p/hsa-miR-365b-3p	0.33	0.0269	0.2620
	hsa-miR-625-3p	0.34	0.0064	0.2325
	hsa-miR-1228-3p	0.35	0.0251	0.2620
	hsa-miR-548at-5p	0.36	0.0153	0.2424
	hsa-miR-125a-5p	0.38	0.0464	0.2965
	hsa-miR-574-5p	0.38	0.0307	0.2692
	hsa-miR-3614-3p	0.39	0.0358	0.2704
	hsa-miR-569	0.39	0.0067	0.2325
	hsa-miR-876-3p	0.40	0.0443	0.2907
	hsa-miR-1238-3p	0.40	0.0467	0.2965
	hsa-miR-1538	0.40	0.0131	0.2424
	hsa-miR-1299	0.40	0.0083	0.2334
	hsa-miR-107	0.40	0.0476	0.2965
	hsa-miR-1281	0.40	0.0472	0.2965
	hsa-miR-3184-3p	0.41	0.0268	0.2620
	hsa-miR-296-5p	0.42	0.0204	0.2537
	hsa-miR-590-3p	0.43	0.0338	0.2704
	hsa-miR-638	0.43	0.0129	0.2424
	hsa-miR-1233-3p	0.43	0.0031	0.2162
	hsa-miR-124-5p	0.43	0.0168	0.2424
	hsa-miR-1321	0.46	0.0155	0.2424
	hsa-miR-1227-3p	0.47	0.0187	0.2424
	hsa-miR-5010-3p	0.47	0.0299	0.2692
	hsa-miR-508-3p	0.48	0.0400	0.2870
	hsa-miR-371b-3p	0.48	0.0125	0.2424
	hsa-miR-4750-5p	0.49	0.0275	0.2625
	hsa-miR-4285	0.49	0.0152	0.2424

**Table 4:** DEMs identified from Cancer (C) vs. Adenomas (B) comparison

	ID	Fold change	*P*-value	FDR
Up-regulated	hsa-miR-5581-3p	3.84	0.0374	0.8256
	hsa-miR-5704	3.82	0.0208	0.8256
	hsa-miR-4463	2.24	0.0250	0.8256
	hsa-miR-3195	2.14	0.0401	0.8256
Down-regulated	hsa-miR-3655	0.25	0.0497	0.8256
	hsa-miR-198	0.29	0.0101	0.8256
	hsa-miR-4506	0.29	0.0297	0.8256
	hsa-miR-130b-3p	0.29	0.0108	0.8256
	hsa-miR-4483	0.30	0.0047	0.8256
	hsa-miR-1205	0.34	0.0480	0.8256
	hsa-miR-431-5p	0.35	0.0426	0.8256
	hsa-miR-548 k	0.35	0.0216	0.8256
	hsa-miR-99b-3p	0.38	0.0326	0.8256
	hsa-miR-4652-3p	0.39	0.0343	0.8256
	hsa-miR-30b-3p	0.42	0.0396	0.8256
	hsa-miR-371b-3p	0.47	0.0303	0.8256
	hsa-miR-135a-3p	0.50	0.0436	0.8256

**Fig. 2:**
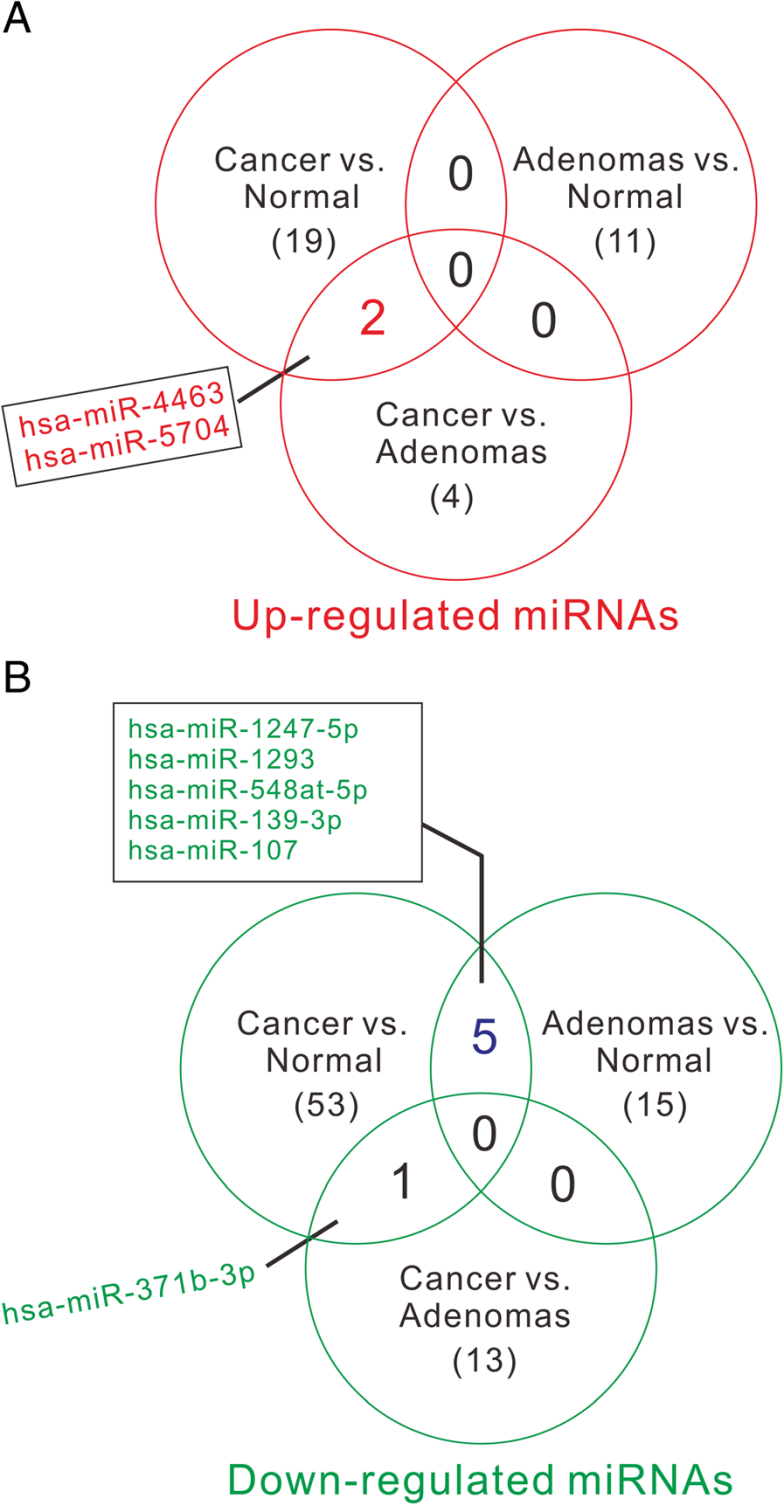
Venn diagrams showing the overlap of DEMs identified from each of the three comparisons: Cancer vs. Normal, Adenomas vs. Normal, and Cancer versus Adenoma, respectively. **a** The overlap of up-regulated DEMs; **b** the overlap of down-regulated DEMs

As shown in Fig. 2, three DEMs (two up-regulated, miR-4463 and miR-5704; one down-regulated, miR-371b-3p) identified from Cancer versus Normal comparison were included in the list of DEMs identified from Cancer versus Adenomas comparison. These three miRNAs may be diagnostic biomarkers for colon cancer. Five down-regulated miRNAs (miR-1247-5p, miR-1293, miR-548at-5p, miR-107, and miR-139-3p) identified from Cancer versus Normal comparison were included in the list of DEMs identified from Adenomas versus Normal comparison. These five serum miRNAs decreased in patients with precancerous polyps and patients with colon cancer, which may be useful for the detection of polys and the prevention of colon cancer. The potential target genes of these DEMs may involve in response to DNA damage, cell apoptosis, cell proliferation, protein tyrosine kinase activity and transcriptional misregulation in cancer (Additional file 1: Table S2 and S3).

### Validation of miRNA differential expression by qRT-PCR

**Fig. 3:**
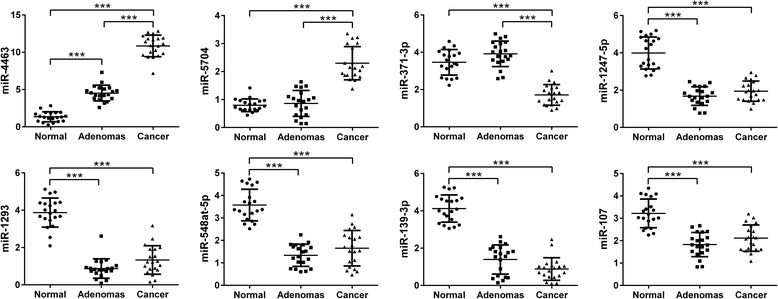
MicroRNA validation by qRT-PCR analysis. Serum levels of miR-4463, miR-5704, miR-371b-3p, miR-1247-5p, miR-1293, miR-548at-5p, miR-107, and miR-139-3p in healthy normal subjects (Normal, *n* = 20), patients with with colon adenoma (Adenomas, *n* = 20), patients with colon cancer (Cancer, *n* = 20). Expression levels of the miRNAs are normalized to cel-miR-39. Statistically significant differences were determined using one-way-ANOVA test. *P < 0.05, ***P < 0.001

MiR-4463, miR-5704, miR-371b-3p, miR-1247-5p, miR-1293, miR-548at-5p, miR-107, and miR-139-3p were included in the validation analysis (Fig. 3). Our validation cohort included a total of 20 patients with colon adenomas, 20 patients with colon cancer and 20 healthy controls. Statistical analysis led to the validation of all detected miRNAs by using this independent cohort. Hence miR-4463 and miR-5704 were confirmed to be significantly up-regulated in colon tumors as compared to colon adenomas or healthy controls, whereas miR-371b-3p was confirmed to be down-regulated. The other five miRNAs were confirmed to be significantly down-regulated in colon adenomas and cancers as compared to healthy controls.

## Discussion

miRNAs are involved in cell differentiation, proliferation and apoptosis [5]. The dysregulated microRNA expression has been reported in colon adenomas and colon cancer [8–11, 18]. In this study, miRCURY LNA™ microRNA Array system was used. This system covers all human microRNAs annotated in miRBase 18.0 and 25 miRPlus™ human microRNAs not found in miRBase. We demonstrate distinct differences in the expression profile of serum miRNA from patients with colon adenomas, patients with colon cancer patients and healthy subjects.

We identified 72 DEMs between patients with colon cancer and healthy subjects, some of which were consistent with previous studies in tissue or blood samples, such as miR-10a-3p [17, 32, 33], miR-125a [34], miR-139-3p [35, 36] and miR-590-5p [35]. Although not reported in colon cancer, some DEMs, such as miR-302c-3p [26], miR-487a [30] miR-638 [31], have been found involved in the survival, invasion or chemoresistance of several cancer cell lines. We also identified 26 novel significant DEMs in colon adenoma samples when compared with healthy subjects, some of which have previously reported as tumor-related miRNAs, such as miR-22 [37], miR-620 [38], miR-765 [39], and miR-1247 [40].

More importantly, three miRNAs which were significantly different between cancer cases and controls, namely miR-4463, miR-5704, and miR-371b-3p, which was validated by RT-PCR. MiR-4463 and miR-5704 was significantly up-regulated and miR-371b-3p was down-regulated in colon tumors as compared to colon adenomas or healthy controls. But here we found miR-5704 and miR-371b-3p had no significant difference between adenomas and healthy controls. This results might indicate that the miR-4463 was a more sensitive index to predict cancer progression. Target prediction.

Five miRNAs (miR-1247-5p, miR-1293, miR-548at-5p, miR-107 and miR-139-3p) was found decreased in both adenomas and cancer cases compared to normal controls, which may be important for the development of cancer, and useful for the detection of polyps and the prevention of colon cancer. These five miRNAs was down-regulated stably in the adenoma and tumor stage, which indicated that it might be good indexes to judge or predict tumorigenesis or recrudescence. These miRNAs may be specific diagnostic biomarkers for colon cancer and further clinical investigations are needed. What’s important, it was the first time to report that miR-4463, miR-5704, and miR-371b-3p, miR-1247-5p, miR-1293, miR-548at-5p was associated with colon cancer, excepting miR-107 [41, 42] and miR-139-3p [43, 44].

The expression changes of these 8 DEMs were confirmed by qRT-PCR using an independent cohort. Preliminary analysis revealed that the target genes of these 8 DEMs may regulate various cellular processes, such as response to DNA damage, cell apoptosis, cell proliferation, protein tyrosine kinase activity, and transcriptional misregulation in cancer. Further in vitro experiments in cell lines may help elucidate the functions of these miRNAs.

## Conclusion

In summary, we discovered 8 circulating miRNAs associated with colon adenomas and colon cancer, and these miRNAs may potentially serve as noninvasive screening biomarkers for colon cancer.

## Additional file

Additional file 1: Target genes of DEMs prediction method. **Table S1.** Primer sequences for qRT-PCR. **Table S2.** Predicted targets of miRNAs. **Table S3.** Functional analysis of target genes. (DOCX 24 kb)

## Abbreviations

DEMs: differentially expressed miRNAs
miRNAs: microRNAs
qRT-PCR: quantitative RT-PCR
TNM: Tumor node metastasis
