# Supplementary material for: Serum MicroRNA profile in patients with colon adenomas or cancer
Source: BMC Med Genomics. 2017 Apr 20;10:23. doi: 10.1186/s12920-017-0260-7 (PMC5399348; doi:10.1186/s12920-017-0260-7)
Supplement: Additional file 1: — Target genes of DEMs prediction method. Table S1. Primer sequences for qRT-PCR. Table S2. Predicted targets of miRNAs. Table S3. Functional analysis of target genes. (DOCX 24 kb) [file 12920_2017_260_MOESM1_ESM.docx]

Additional file 1

**Supplementary methods**

Target genes of DEMs were predicted using v6.2 TargetScan Human Database. Functional analysis of the target genes was performed by DAVID Bioinformatics Resources (<https://david.ncifcrf.gov/>) [[11](#_ENREF_11)] as previously described. A statistical significance of *P*<0.05 was set as cut-off.

**Table S1. Primer sequences for qRT-PCR**

| **ID** | Forward Primer (5' to 3') | Reversed Primer(5' to 3') |
| --- | --- | --- |
| miR-4463 | GCAGGAGACTGGGGTG | GTCCAGTTTTTTTTTTTTTTTGGC |
| miR-5704 | CAGTTAGGCCATCATCCCA | GGTCCAGTTTTTTTTTTTTTTTGCATA |
| miR-371b-3p | GCCCCCACAGTTTGAG | GTCCAGTTTTTTTTTTTTTTTGCAC |
| miR-1247-5p | CCCGTCCCGTTCGT | GTCCAGTTTTTTTTTTTTTTTCCG |
| miR-1293 | GTGGGTGGTCTGGAGA | CCAGTTTTTTTTTTTTTTTGCACA |
| miR-548at-5p | CGCAGAAAAGTTATTGCGGTT | GTCCAGTTTTTTTTTTTTTTTAGCCA |
| miR-107 | GCAGAGCAGCATTGTACAG | GGTCCAGTTTTTTTTTTTTTTTGATAG |
| miR-139-3p | CGCGGCCCTGTTG | GGTCCAGTTTTTTTTTTTTTTTACTC |

**Table S2. Predicted targets of miRNAs**

| **ID** | **Target genes** |
| --- | --- |
| miR-4463 | UHMK1,C5ORF22,GNL3L,RYBP,SEPT8,UQCRH,POTED,IFNLR1,G3BP1,CHAC1, CAD,BMP2,ASXL1,HES7,SPATA6,GPR20,MID1IP1,ZNF736,TBC1D19,RTKN, SH3BP5,HAT1,TSKU,CDKN1B,FRK,INTU,BTLA,KRTAP5-6,PTAFR,THAP1, TRIM72, SFT2D2, KY,ZNF772,KLRD1,RBM43,MLEC,INIP,FKBP15,SERPINH1, CC2D2A,PURB,HIF1A,WNK3,PPIC,ARGLU1,ENSA,TBC1D4,SLC6A9,FAM206A,CAV1,GPR173,MAP1B,GFPT1,C9orf64,KIAA0586,IPP,RSBN1L,BRMS1L,SLC10A6,SOX12,THAP6,LONRF3 |
| miR-5704 | RPL37A,C17orf96,KCNH5,ZNF514,CTNNA3,ENAM,ECHDC3,PLSCR1,TRAPPC6B,TEX261,ZBTB45,TMEM64,TAGLN2,CASP16,TRADD,LONRF3,SLC1A5,UQCR10,AGBL3,ZNF607,CLCN6,DPY19L1,SLC36A1,GJB2,SH3BGRL,PRMT3,SNRPB,C10orf2,OCLN,KIAA1804,ZNF35,CADM2,SRRD,RBM3,DSN1,PGRMC1,ZBTB8B,TMEM216,CD3EAP,NDUFA7,SLU7 |
| miR-371b-3p | SHOC2,GDF11,BTG2,CDCA8,CRKL,ZNF75A,COX15,CHML,EVX2,FLT1,PRICKLE4,STAT3,TMEM231,FBXO6,NCAPD2,BNIP2,AFF1,C1orf64,SMC1A,SAR1B,PPP2R5D,AREL1,OIP5,SYNM,TPM3,MAPK1,NSA2,ATXN1,ANKRD52,TRIM10,NUDT16,PHACTR2,ZC3H12B,SPN,MAP3K9,UEVLD,CDK9,REL,IGFBP3,TBC1D21,SEPT5,CTC1 |
| miR-1247-5p | RPL35A,ALDH16A1,WAC,SOX9,EFHD2,TESK2,TESK2,TESK2,DVL3,HPR,ARL6IP1,EIF2B5,TCF20,ATXN1,CYP2B6,CYBRD1,C19orf68,RIMS3,ADRA2B |
| miR-1293 | GCN1L1,FZD6,CHAC1,MAPK6,ITGA2,TIMP1,NLRP6,CSNK1E,ARMCX6,RMI1,PACSIN1,UBTF,TPM3,SNAPIN,SETD5,RNPS1,RNF121,PHF19,KLF16,HIC2,CPSF7,APEX1,TRAF6,C15orf52,SLC35C2,CYP4A22,CYP4A11,HIST2H3C,HIST2H3A,CCDC64,SESTD1,DNLZ,MRRF,PRRT2,TAOK1,NFIX,NFIC,BCL7A,METTL14,HIST1H2AG,SREK1IP1,SGMS2,HIST2H2AA4,HIST2H2AA3,HIST1H3H,HIST1H3B,HIST1H2BF,HIST1H2AD,CRCP,HIST2H2AC,VEGFA,MAGI3,HIST3H3,HIST3H2BB,HIST1H2AE,HIST1H1E,MTRNR2L7,ALOX15,ULK1,HIST2H2BE,CNNM4,KPNA6,TPCN2,TOB2,C12orf49,ZNF703,ZNF445,COL4A3BP,SPATA5,CEP97,CAPZA2,PPP1R15A,FAM83F,DSN1,PMPCA,C10orf76,PLEKHA1,ZNF107,PCNP,ABI2,LRTOMT,LLGL1,INMT,ZNF641,KLHL36,TRAPPC3L,NFATC2IP,BFAR |
| miR-548at-5p | ZFP36L1,STX6,IMPDH1P11,CBX5,RNF11,PCBP1,TRIM24,ZNF607,OXGR1,NACC2,UCP1,MON1B,PEX5L,LRRC63,ZNF579,NT5DC1,TMF1,TMEM248,PRDX3,GPBP1L1,DONSON,SPIC,FBLN2,CCDC117,SLC38A2,PTBP2,PMEPA1,PHOX2B,NAA50,MAP4K5,INIP,ELOVL6,DDX3X,ANP32E,ANKRD28,LYRM2,MDM2,EGLN1,NDRG1,TRIM66,RFTN2,SLC7A5P2,ZBTB10,USP48,RNF6,GFPT1,ZNF138,TTC8,UBE2A,TAF13,SUPT7L,ROBO1,RCAN2,HOXA9,DHX33,TMEM98,TWF1,GINM1,RMI2,PPWD1,SON,ITGB1,CEP97,YWHAQ,LSM11,DDX21,TP73,CREBL2,SCYL3,FAM208A,DHX36,TAF8,ZNF70,VBP1,WASL,STK38,RAB14,PCGF5,DGS2,DCLK3,LMBRD2 |
| miR-139-3p | MTDH,SOX4,ZBTB7A,EHD3,ALOX15,ZNF585B,FAM162A,ZNF589,TBX20,HLAA,FOXC1,MRPL9,VWA1,SNRNP27,SMIM7,SLC5A6,KLHL21,DNAL1,CCDC117,B2M,ATP5A1,LRRC45,SYT7,SLC38A4,GFRA1,FAM83H,GDF5OS,HAND2,SSBP2,THY1,GZMM,BARHL1,LRPPRC,RASD1,LIMD2,EN1,UCP2,PDGFRA,UBE2G1,CCDC71L,URGCP,MRPS24,SMCR7L,ATG2A,SUSD1,FN3K,HNRNPC,ZNF486,PAX5,HIST1H2AH,DDX19A,SORBS2,NLGN4X,BMPR1A,AMDHD2,C14orf180,ZNF550,TMEM170A,CHEK1,HIST1H2BN,ZSWIM7,LRTOMT,KRT80,PNPO,WDR13,BCAR1,ORAI2,MTHFD1,FAM60A,FAM102B,CCDC36,ALG14,PKIA,TUBD1,RBM8A |
| miR-107 | PLAG1,BACE1,CDK6,MYB,VEGFA,HIF1A,ARNT,CDCA4,CCNE1,RAB1B,CRKL,FBXW7,GRN,NFIA,DICER1,DAPK1,KLF4,PTEN,AXIN2,CYP2C8,CHRM1,NOTCH2,SAT1,GUCD1,AGO2,RPL27,NINJ1,METRNL,AGO3,INSIG1,IER3IP1,ZDHHC4,AGO1,CSNK1G2,MRPL2,ADD2,MRPL12,C21orf58,GANAB,TUBA1B,SNX8,DHX16,ATXN1L,TUBB,BABAM1,PCSK5,RPAIN,PHGDH,RPSA,ZADH2,TCF19,FAM207A,TM9SF1,COPS7A,PSMB6,WDR6,GNS,GPR89B,TMEM87A,PRKCE,FOXO1,CDK8,CAV1,LATS2,SNCG,OPRM1,JAK1,IL6,CNNM2,PLEKHA1,ZDHHC16,TARBP2,MTFR1L,DYRK2,FCF1,ARIH1,SNTB2,VPS4A,C16ORF72,RPS6KB1,SOWAHC,UBR3,YWHAH,NUP50,B3GNT2,ACTR2,ACVR2B,SLAIN2,CALU,USP42,PLEKHF2,OGT,TNRC6B,FGFRL1,CREBRF,LCOR,SLC30A7,PAFAH1B2,CDADC1,HIC2,CAB39,FGF2,FZD6,ZNF449,CDC42SE2,NUCKS1,SETD1B,PPP2R5C,FAM103A1,SLCO3A1,MTMR3,RAB10,ITGA2,PIK3R1,VCAN,CAPZA2,RAD51,SALL4,ERN1,ALDH3B1,EFTUD2,IDH3A,DOCK11,ZBTB38,UBE2Q1,TM4SF1,TBPL1,STX6,STK38,RNF168,REL,RAD21,MT1E,JOSD1,GOLGA8B,FURIN,MPLKIP,SEMA6A,CD180,RPSAP58,ORC4,ODF2L,ZNF606,DNAJA1,ABCF2,L2HGDH,POLD3,SREK1,RBBP6,IGSF3,FAM98A,CSNK1G3,ABL2,ATG12,RCC1,MRPL51,YTHDC1,SRSF1,SMARCE1,RIMS3,PRR14L,PPIG,PHKA1,PDZD8,KIF23,GPCPD1,G3BP2,EN2,ELK4,DHX33,ZNF273,MCM7,TRIQK,PPP2CA,MAP3K7,PAWR,CSNK2A1,TRIM35,NNT,SLC2A3,PPP6R3,PPIL1,PER1,MYBPC1,EI24,MPDU1,ENPP2,DST,FOXC1,NPY4R,GNAT1,BAZ2A,ZNF585B,GSG1,LRIF1,TMEM170A,MIS18BP1,CCDC83,LIN7C,DECR1,SALL1,RUNX1T1,PURB,PPP6C,PNISR,PAG1,NUMB,HCFC2,EML4,EIF1AX,ASH1L,AMOT,HDDC2,RSL1D1,ENTPD1,FAM229B,ZNF100,DMPK,SSU72,DNAJC10,ZBTB10,YRDC,YIPF6,YAF2,UBE2B,TM7SF3,TJP1,TBRG1,TAF13,SUN2,RRAGC,RACGAP1,OTUD7B,LUC7L,DUSP14,CLIP1,CDV3,ATP13A3,ATG14,ARGLU1,TWF1,HNRNPA2B1,CDK1,ZNF680,SLC28A1,DEPDC1B,CCNT1,TGFBR3,POLDIP2,LBR,GNG12,CPEB3,PPP1R16B,TSC22D2,TTLL5,SMARCA5,HPRT1,NUS1,N4BP1,C9orf62,GABRB1,NACC2,GPRC5A,TULP4,BTLA,FAM9C,GCC1,KDELR1,FAM49A,RS1,GLP2R,TK1,RPS24,ANKFY1,CKMT1A,ZCCHC14,ADORA3,CYSLTR2,SYNRG |

**Table S3. Functional analysis of target genes**

| **ID** | **Target genes** | **Function** | ***P* Value** |
| --- | --- | --- | --- |
| **miR-4463** | BMP2, CDKN1B, CHAC1, HES7 | Notch signaling pathway | 0.00603 |
|  | CAV1, BMP2, HIF1A | positive regulation of endothelial cell proliferation | 0.02191 |
|  | SH3BP5, CAV1 | negative regulation of protein tyrosine kinase activity | 0.03483 |
| **miR-5704** | SNRPB, SLU7 | small nuclear ribonucleoprotein complex | 0.03220 |
|  | PRMT3, SNRPB | protein methylation | 0.04952 |
| **miR-371b-3p** | FLT1, REL, CDK9, AFF1, IGFBP3 | Transcriptional misregulation in cancer | 0.00039 |
|  | MAPK1, BTG2, CTC1 | cellular response to DNA damage stimulus | 0.01980 |
|  | MAPK1, FLT1, STAT3 | signaling pathway | 0.02344 |
| **miR-1247-5p** | DVL3, SOX9 | cochlea morphogenesis | 0.01697 |
| **miR-1293** | HIST2H3A, PHF19, HIST1H3B, HIST2H3C, HIST1H3H,SKOR1,VEGFA, KLF16, MNT, NFIX, TRAF6, NFIC | negative regulation of gene expression, epigenetic | 0.00012 |
|  | NLRP6, ULK1, ULK2, TPCN2 | regulation of autophagy | 0.00232 |
| **miR-548at-5p** | ZNF275, GPBP1L1, ZBTB10, E2F7, TMF1, PCGF5, DDX3X, HOXA9, ZNF579, SUPT7L, DHX36, SPIC, ZNF607, ZNF70, CREBL2, PHOX2B, NACC2, TAF8, TEAD1, WHSC1, TRIM24, PROX2, SIRT1, TP73, FAM208A, ZNF138, TAF13, HNRNPUL1, FOXC1, WASL | Transcription regulation | 0.00005 |
|  | TAF13, MDM2, RMI2, TRIM24, TP73 | regulation of signal transduction by p53 class mediator | 0.00764 |
|  | IGF1R, SON, XIAP, DDX3X, MDM2, PRDX3, AGO4, SIRT1, TMF1 | negative regulation of apoptotic process | 0.00886 |
|  | MSH6, UBE2A, RAD51AP1, TAOK1, INIP, SIRT1 | DNA repair | 0.01606 |
|  | PHOX2B, NACC2, E2F7, NDRG1, TRIM24, ITGB1, TP73 | negative regulation of cell proliferation | 0.04337 |
| **miR-139-3p** | HAND2, BARHL1, TBX20, PAX5, SOX4, FOXC1 | transcription from RNA polymerase II promoter | 0.03712 |
|  | MTHFD1, HAND2, SOX4, FOXC1 | heart development | 0.03973 |
| **miR-107** | IL6, RUNX1T1, ITGA2, FOXO1, CDK6, GNG12, PTEN, RAD51, DAPK1, FZD6, ARNT, CCNE1, CRKL, HIF1A, VEGFA, JAK1, AXIN2, FGF2, PIK3R1 | Pathways in cancer | 0.00017 |
|  | IL6, PPP2R5C, ITGA2, RPS6KB1, CDK6, GNG12, PTEN, CCNE1, YWHAH, PPP2CA, CHRM1, VEGFA, JAK1, MYB, FGF2, PIK3R1 | PI3K-Akt signaling pathway | 0.00103 |
|  | MAP3K7, PPP2CA, PPP2R5C, FOXO1, RPS6KB1, CAB39, RAB10, PIK3R1 | AMPK signaling pathway | 0.00565 |
|  | CDK1, FOXO1, RPS6KB1, PAWR, PRKCE, PTEN, DAPK1, RRAGC, MAP3K7, NOTCH2, SEMA6A, EI24, TRIM35, RAD21, CSNK2A1, ZDHHC16, PPP2CA, OGT | apoptotic process | 0.00574 |
|  | TARBP2, IL6, CPEB3, ITGA2, RPS6KB1 | positive regulation of translation | 0.00942 |
|  | GNAT1, CDK1, CDV3, MCM7, CHRM1, INSIG1, ITGA2, TCF19, FOXC1, AXIN2, PTEN, FURIN, PURB | cell proliferation | 0.01007 |
|  | IL6, FOXO1, RPS6KB1, OGT, PRKCE, PTEN, PIK3R1 | Insulin resistance | 0.01186 |
|  | RPS6KB1, CAB39, PTEN, PIK3R1, RRAGC | mTOR signaling pathway | 0.01886 |
|  | CCNE1, CDK1, RAD21, MCM7, YWHAH, ORC4, CDK6 | Cell cycle | 0.02209 |
|  | CRKL, HIF1A, VEGFA, PIK3R1, ARNT | Renal cell carcinoma | 0.02740 |
|  | VEGFA, JAK1, CDK6, PIK3R1, RAD51 | Pancreatic cancer | 0.02740 |
|  | CCNE1, CDK1, EI24, CDK6, PTEN | p53 signaling pathway | 0.03021 |
